# Chromoblastomycosis-Leprosy Co-Infection in Central West Brazil. Presentation of Three Cases and Literature Review

**DOI:** 10.1007/s11046-022-00646-5

**Published:** 2022-06-28

**Authors:** Armando Guevara, Vânia Aparecida Vicente, Bruna Jacomel F. de Souza Lima, Andréia Ferreira Nery, Ferry Hagen, Rosane Christine Hahn

**Affiliations:** 1 Laboratory of Mycology/Research, Faculty of Medicine, Federal University of Mato Grosso, Cuiabá, Mato Grosso Brazil; 2 Microbiology, Parasitology and Pathology Post-Graduation Program, Department of Pathology, Federal University of Paraná, Curitiba, Paraná Brazil; 3 Júlio Muller University Hospital - Ebserh, Federal University of Mato Grosso, Cuiabá, Mato Grosso Brazil; 4 Department of Medical Mycology, Westerdijk Fungal Biodiversity Institute, Uppsalalaan 8, 3584CT Utrecht, The Netherlands; 5 Institute for Biodiversity and Ecosystem Dynamics, University of Amsterdam, Amsterdam, The Netherlands; 6 Department of Medical Microbiology, University Medical Center Utrecht, Utrecht, The Netherlands

**Keywords:** Chromoblastomycosis, Leprosy, Co-infection, Neglected diseases, Aging

## Abstract

Chromoblastomycosis and leprosy are chronic diseases with high prevalence in tropical and subtropical regions. Brazil is one of the countries with the highest incidence and prevalence for both diseases, however, reports of co-infections are scarce. The aim of this study was to describe three cases of chromoblastomycosis-leprosy co-infection in patients from Mato Grosso state, Brazil. A review of chromoblastomycosis-leprosy co-infection was performed of English, Portuguese and Spanish publications in LILACS, SciELO, PubMed and Web of Science databases using the descriptors (chromoblastomycosis OR cromoblastomicose OR cromoblastomicosis) AND (leprosy OR hanseníase OR lepra), without time period delimitation. Nineteen cases were included, 16 cases were published in 11 articles, plus the three cases reported in the current study. Most reported coninfection cases came from Brazil. Majority of the patients were male with a mean age of 52.2 years. Farmer was the main occupational activity reported. In 12 patients, the clinical signs and symptoms of leprosy started first. No contacts with patients affected by leprosy, armadillos or history of injuries at the anatomical site of chromoblastomycosis lesions were reported. Five leprosy patients who received steroid treatment for leprosy reactions or neuropathies, were diagnosed with chromoblastomycosis during immunosuppressive therapy. Four cases (21.1%) were reported among the elderly patients. Co-infections in patients with chromoblastomycosis or leprosy are uncommon, but the possibility should always be considered, especially if the patient is undergoing immunosuppressive treatment or is elder.

## Introduction

Chromoblastomycosis and leprosy are highly prevalent diseases in tropical and subtropical regions. Brazil, China, Madagascar, Mexico, and Venezuela are the countries mainly affected by chromoblastomycosis, while leprosy is predominantly reported from Brazil, India and Indonesia [1–7]. Both diseases are characterized by social, cultural, economic, lifestyle, poverty and inequality factors, in addition to the host its genetic susceptibility to develop leprosy [8–11].

Chromoblastomycosis is a chronic granulomatous disease produced by dematiaceous fungi that affects the skin and subcutaneous cellular tissue. It usually starts with the traumatic inoculation of conidia or fragments of hyphae into the patient's tissues. The most frequent etiological agents belong to the genera *Fonsecaea* and *Cladophialophora.* This disease mainly affects males in their productive age, but there are reports of very young patients and elderly with a reported range of 2–99 years of age. Patients have often occupations related to plant-and-soil activitie, such as farmers, gardeners, lumberjacks, and sellers of agricultural products. Hence, chromoblastomycosis is considered an occupational and implantation disease [1, 8, 12, 13].

Leprosy is a bacterial disease caused by *Mycobacterium leprae* and *Mycobacterium lepromatosis* that both affect the skin and peripheral nerves, and potentially can spread to other organs. This disease is observed in both sexes and all age groups; it is transmitted through close contact between patients and susceptible people, possibly through upper respiratory tract secretions. If leprosy is not well diagnosed and treated, it can produce disability and deformities with a detrimental impact on the patients personal and social life due to the stigma related to the disease [2, 3, 10].

Few cases of chromoblastomycosis-leprosy co-infection have been described. Here, we describe three of such cases from Mato Grosso state, Brazil, and review the previously reported cases.

## Case Reports

### Case 1

**Fig. 1:**
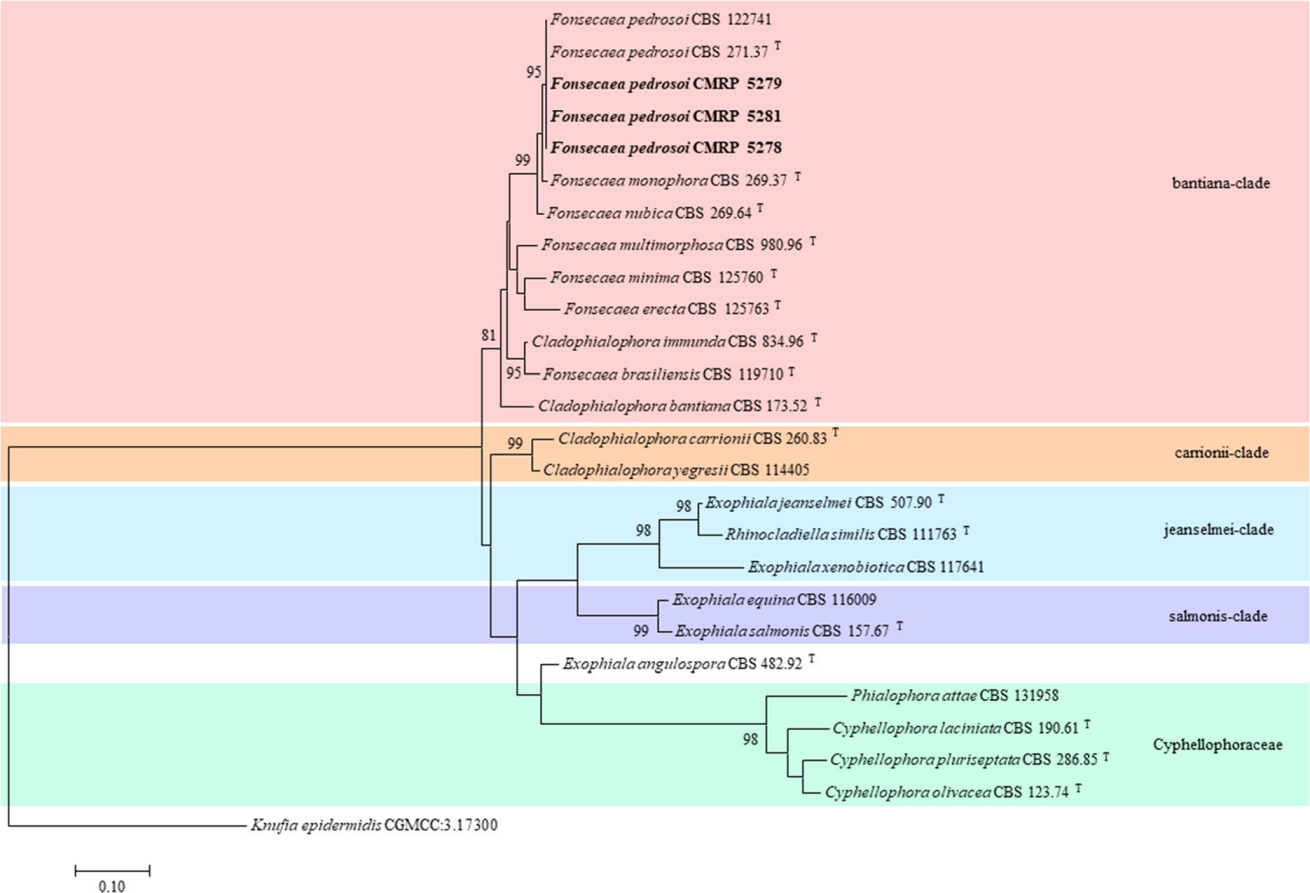
Molecular identification of the three chromoblastomycosis cases. Phylogenetic tree based on combined analysis (ITS and BT2a) constructed with maximum likelihood implemented in MEGA v7.0.26 using Tamura-Nei with gamma variation (TN93+G) model. Bootstrap values above 80, from 1000 resampled datasets, are shown along the branches. The clinical strains from the here reported chromoblastomycosis cases are indicated in bold. T indicates type strain. The *Knufia epidermidis* CGMCC:3.17300 strain was taken as outgroup

Female patient, 91 years old, retired, residing in Cuiabá, capital of the Mato Grosso state, Brazil, with a history of systemic arterial hypertension, osteoporosis, cholecystectomy and gastrectomy for gastric cancer 30 years earlier. The patient presented to the Clinic of Infectious and Tropical Diseases of the Júlio Müller Universitary Hospital (CDIT-HUJM), Cuiabá-Brazil, in July 2016, with an itchy, scaly lesion in the posterior region of the left leg that appeared two months earlier, a hypochromic lesion on the right arm, and lesions hyperchromic lesions in the left gluteus and left knee, had developed over the past year. On physical examination, a scaly erythematous plaque with regular edges and ill-defined contours, hyperkeratotic with blackened dots on the surface, measuring 6 × 4.5 cm, on the posterior surface of the left leg was observed. Slightly desquamative hypochromic macule on the right arm and macules with a hypochromic center and reddened edges were observed on the left gluteus, lower 1/3 of the left thigh, left knee and in the plantar regions. The patient had dysautonomia in the feet and arthrosis in the toes. She had no neuritis. The thermal sensitivity test in the left thigh region showed hypoesthesia to hot/cold in the lesion. There was no history of trauma at the wound sites or contact with patients with leprosy or armadillos. Skin biopsies were collected from the lesions on the left leg and left gluteus, which were sent for histopathological and mycological examination. The histopathology of the left leg sample reported skin with acanthosis, parakeratosis, granulation tissue, epithelioid granuloma formation, with no evidence of infectious agents. Presence of intermingled eosinophils. Absence of neoplasm. Microscopic examination with a 20% KOH-treated sample—taken from the left leg—showed the presence of muriform cells. The Sabouraud dextrose agar culture yielded a melanized fungus, identified by micromorphology as *Fonsecaea* species. The strain was subjected to (partly) sequencing of the internal transcribed spacer (ITS) and *β*-tubulin gene, as previously described [14, 15]. Raw sequence data was manually checked and corrected BioEdit Sequence Alignment Editor v7.2.5. Alignment, visual data inspection, and phylogenetic reconstruction were done in MEGA v7.0.26, resulting in the identification of the strain as *Fonsecaea pedrosoi* (Fig. 1, Table 1). The strain was deposited, under accession number CMRP 5278, at the Microbiological Collections of Paraná Network/Brazil (https://www.cmrp-taxonline.com/). The skin biopsy of the lesion on the left buttock showed a slight dermal perivascular lymphocytic infiltrate, with no other alterations. The smear to detect acid-fast bacilli was negative. The patient was diagnosed with chromoblastomycosis, based on laboratory results, and borderline leprosy due to her clinical characteristics. In November 2016, replacement treatment was indicated for leprosy (rifampicin, ofloxacin, clofazimine) due to the risk of myelotoxicity. In December 2016, she presented with clearing of the macules in the left gluteus and left knee and neuritis in the ulnar and fibular nerves. She was treated for leprosy for 1 year, until September 2017. In January 2018 she had a type I leprosy reaction (reverse reaction) treated with prednisone for 45 days. In December 2016, she started treatment for chromoblastomycosis that consisted of itraconazole 200 mg/BD and repetitive cryotherapy with progressive improvement of the lesion at last follow-up in January 2020.

**Table 1:** Background information of reference strains and isolates from human subcutaneous infection evaluated in this study

Name	Strain no.	Cross-reference no.	Source/Geographical origin	GenBank accession number ITS and BT2a
*Cladophialophora bantiana*	CBS 173.52 (T)	CBS 100433	Brain abscess, man/USA	EU103989; XM_016765884
*Cladophialophora carrionii*	CBS 260.83 (T)	CDC B-1352, FMC 282	Skin lesion, man/Unknown	MH861582; EU137175
*Cladophialophora immunda*	CBS 834.96 (T)	dH 21287	Subcutaneous mycosis, man/Georgia, Atlanta, USA	NR_111283; EU137203
*Cladophialophora yegresii*	CBS 114405	UNEFM SgSr3	*Stenocereus griseus*, cacti/Venezuela	NR_111284; EU137209
*Cyphellophora laciniata*	CBS 190.61 (T)	dH 15498	Skin, human/Switzerland	NR_121335; JQ766329
*Cyphellophora olivacea*	CBS 123.74 (T)	–	Wet wallpaper/Kiel-Kitzeberg, Germany	NR_156258; KC455231
*Cyphellophora pluriseptata*	CBS 286.85 (T)	–	Fallen leaf *Zostera marina*/Netherlands	NR_111431; KC455225
*Exophiala angulospora*	CBS 482.92 (T)	–	Potable water/Japan	NR_111625; JN112426
*Exophiala equina*	CBS 116009	dH 13221, F1090	Galapagos tortoise/Chicago, USA	KF928433, KF928561
*Exophiala jeanselmei*	CBS 507.90 (T)	dH 15933, ATCC 34123	Man/Uruguay	NR_111129; EF551501
*Exophiala salmonis*	CBS 157.67 (T)	BMU 00834	Trout, brain/Canada	NR_121270; JN112499
*Exophiala xenobiotica*	CBS 117641	UTHSC 99–791	Knee, cyst/USA	DQ182591, DQ182575
*Fonsecaea brasiliensis*	CBS 119710 (T)	dH 16818	Crab, mangrove/Sergipe, Brejo Grande, Brazil	JN173784; JN368478
*Fonsecaea erecta*	CBS 125763 (T)	dH 20513	Thorn, Japecanga (Smilacaceae)/Bacabeira, Maranhão, Brazil	KC886414; KF155221
*Fonsecaea minima*	CBS 125760 (T)	dH 20463	Palm leaf (*Orbignya speciosa*)/Icatu, Maranhão, Brazil	NR_156259; KF155222
*Fonsecaea monophora*	CBS 269.37 (T)	dH 12659	Chromoblastomycosis lesion/South America	NR_131280; EU938547
*Fonsecaea multimorphosa*	CBS 980.96 (T)	NCMH 1412	Phaeohyphomycosis, cat/Queensland, Australia	NR_111612; HQ681121
*Fonsecaea nubica*	CBS 269.64 (T)	dH 15656	Chromoblastomycosis lesion/Cameroon	NR_111333; EU938574
*Fonsecaea pedrosoi*	CBS 271.37 (T)	dH 15659	Chromoblastomycosis lesion/South America	NR_130652; EU938559
*Fonsecaea pedrosoi*	CBS 122741	dH 18431	Chromoblastomycosis lesion/Mexico	EU938589; EU938570
***Fonsecaea pedrosoi***	**CMRP 5281**	**6960**	**Human subcutaneous infection/MT, Brazil**	**OK157453; OK247620**
***Fonsecaea pedrosoi***	**CMRP 5278**	**5356**	**Human subcutaneous infection/MT, Brazil**	**OK157451; OK247618**
***Fonsecaea pedrosoi***	**CMRP 5279**	**6042**	**Human subcutaneous infection/MT, Brazil**	**OK157452; OK247619**
*Knufia epidermidis*	CGMCC:3.17300	–	Rock-inhabiting/China	KP174859; KP226560
*Phialophora attae*	CBS 131958	–	Ant/Brazil	KF928463; KF928591
*Rhinocladiella similis*	CBS 111763 (T)	dH 11329, HC-1	Foot lesion/Minas Gerais, Brazil	NR_111244; EF551521

*T* type strain; *CMRP* Microbiological collections of Paraná Network—https://www.cmrp-taxonline.com/; *CBS* CBS culture collection hosted at the Westerdijk Fungal Biodiversity Institute (Utrecht, The Netherlands; https://www.wi.knaw.nl/). The clinical isolates identified in this study are in bold

### Case 2

A 57-years-old male patient, resident of Mirassol d'Oeste, rural area from Mato Grosso state, Brazil, farmer, smoker, with history of paracoccidioidomycosis of the right leg 12 years before, treated with fluconazole for 4 years and itraconazole for 6 years persisting residual lesions, with no others important pathological antecedents. In November 2017 he consulted at CDIT-HUJM due to pain, erythema and progressive increase of the lesion size on his right leg. The physical examination revealed an extensive verrucous lesion on the right leg and foot, covering the entire circumference of the leg, local hyperemia, scaly crusts and loss of the right toenails. There was no history of trauma at the wound sites or contact with patients with leprosy or armadillos. Biopsies were collected for histopathological and mycological tests. Histopathology reported skin exhibiting pseudoepitheliomatous hyperplasia in the epidermis; chronic inflammatory lymphohistiocytic infiltrate in the dermis. Presence of rounded and brownish fungal structures, compatible with chromoblastomycosis. Direct mycological investigation of the sample with 20% KOH was negative, but culture yielded a melanized fungus identified as *F. pedrosoi* (strain accession number CMRP 5279) using the above described molecular identification approach using ITS and *β*-tubulin gene sequencing (Fig. 1, Table 1; [15, 15]). The diagnosis of chromoblastomycosis was made. Treatment with itraconazole 200 mg/day and cryotherapy was indicated. In February 2019, the patient reported paresthesia in both hands and bilateral loss of strength, more pronounced on the right hand that had evolved during the past year. Physical examination revealed thickening of the radial, brachial and posterior tibial nerves bilaterally; hypotrophy of the bilateral thenar region, being diagnosed with pure neural leprosy, multidrug therapy was indicated (rifampicin, dapsone, clofazimine). At follow-up in January 2020, there was no further improvement of the chromoblastomycosis lesions, while treatment for leprosy had to be continued.

### Case 3

A 65-year-old male patient from Rosário Oeste, a rural area central in the state of Mato Grosso, Brazil, farmer, with a history of smoking, peripheral vascular insufficiency, megaloblastic anemia, benign prostatic hyperplasia, bilateral inguinal herniorrhaphy 12 years before, and total gastrectomy for peptic ulcer 25 years earlier. In October 2019, he was admitted to Júlio Müller Universitary Hospital, Cuiabá-Brazil, for treatment of megaloblastic anemia. During hospitalization, he was diagnosed with chronic obstructive pulmonary disease, borderline-dimorphic leprosy and possible chromoblastomycosis/leishmaniasis of the left upper limb. He was referred to the CDIT-HUJM for treatment of leprosy and diagnosis and treatment of the lesion in the left upper limb. Physical examination showed acrocyanosis and five ulcerative lesions in the distal third of the right leg and foot and loss of tactile and painful sensitivity in the region, approximately one year of evolution. Scar lesion on the left elbow, with presence of crust in the lateral region, without secretion or blood, approximately five years of evolution. Presence of multiple hyperchromic macules, paresis and paresthesia in upper limbs. Bilateral thickened visible auricular nerve. No history of trauma to the left elbow, contact with patients with leprosy or armadillos was reported. Replacement treatment for leprosy (rifampicin, ofloxacin, clofazimine) was indicated due to the risk of myelotoxicity. Samples were collected from the elbow lesion for further mycological investigations. The direct observation with 20% KOH showed the presence of muriform cells and the culture yielded a melanized fungus that was subsequently molecularly identified as *F. pedrosoi* using the same molecular identification methodology as applied for the above two cases (strain accession number CMRP 5281; Fig. 1, Table 1; [14, 15]). The patient did not return to the CDIT-HUJM for further follow-up.

## Literature Review Methodology

The search for articles was carried out in August 2020 using the LILACS, SciELO, PubMed and Web of Science databases. For this purpose, Descriptors in Health Sciences (DeCS) and Medical Subject Headings (MeSH) were used in English, Portuguese and Spanish. The descriptors used were: (chromoblastomycosis OR cromoblastomicose OR cromoblastomicosis) AND (leprosy OR hanseniase OR lepra), without date of publication limitation. Only cases of patients with active chromoblastomycosis and leprosy, published in any of the three languages used in the search, which had full text available online or in printed form in Brazilian libraries, were included in the review. Duplicate articles, opinion articles, editorials, and review articles, as well as theses, dissertations, conclusions papers, monographs, books and government documents were excluded. The bibliographic references of the selected studies were examined to identify other publications that were not found in the searches performed in the databases.

For data collection, an electronic database was setup, that included the following variables: authors, year of publication, country where the study was carried out, gender of patients, age, occupation, disease that started first, immunosuppressive treatment prior to the onset of diseases, previous contact with patients with leprosy or armadillos, time of evolution of leprosy until the diagnosis of chromoblastomycosis, classification of leprosy (Ridley-Jopling), type of lesions, sequelae, leprosy reactions, time of evolution of chromoblastomycosis until the diagnosis of leprosy, history trauma at the site of chromoblastomycosis lesions, clinical forms of lesions, disease severity, etiologic agent of chromoblastomycosis. To register the clinical form of chromoblastomycosis lesions, the terms of the old classifications were adapted to the Carrion's classification according to the clinical characteristics described in the reviewed articles. The severity of the disease was classified as previously described [1]. The current nomenclature of the etiological agents of chromoblastomycosis was used to register the (molecularly) identified etiological agents.

## Results

**Fig. 2:**
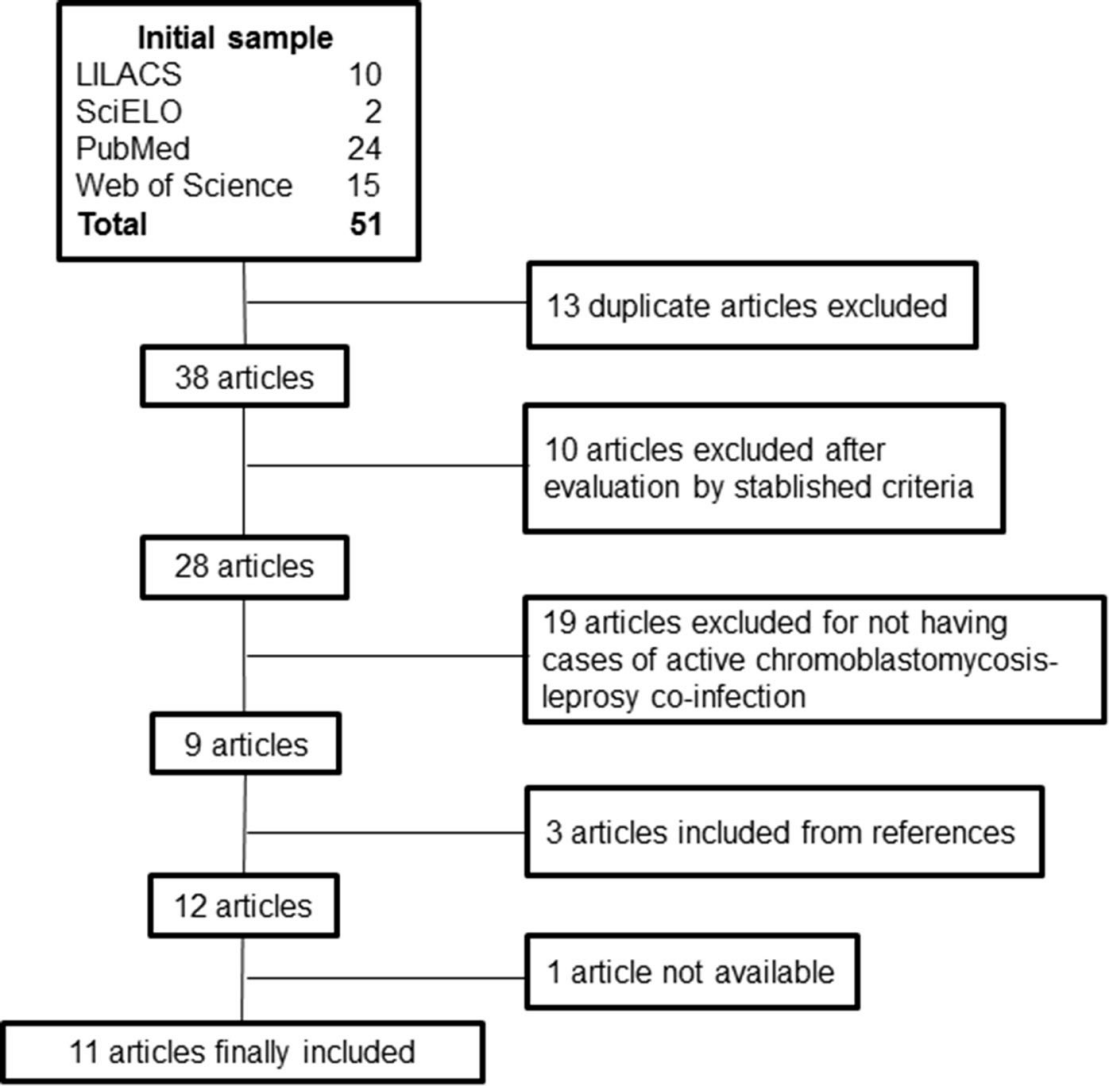
Flowchart diagram of article selection addressing chromoblastomycosis-leprosy co-infection

The initial databases search identified 51 articles. After excluding the duplicates and applying the inclusion criteria, 9 articles with cases of active chromoblastomycosis-leprosy co-infection were selected. Through the manual review of the bibliographic references, 3 more articles were found that contained cases of co-infection. One of the selected articles, published in 1959, was excluded because it was not available for evaluation [16] (Fig. 2). In total, 19 cases were included, 16 cases published in the 11 selected articles and the three cases reported in the current study.

Brazil was the main country reported, with 15 cases, followed by India and Japan (Table 2). Most patients were male with a mean age of 52.2 years (SD ± 20.074 years; median 50 years; range 24–91 years; data available for 11 patients). The main occupation reported was farmer. In 12 patients, the clinical signs and symptoms of leprosy had first started, or this disease had been diagnosed previously. While for 7 patients, chromoblastomycosis was started first. Of the 12 patients in whom leprosy started first, only 4 reported the time of evolution of this disease to the diagnosis of chromoblastomycosis. The average time of evolution was 6 years (SD ± 8.042 years; median 2.5 years; range 1–18 years). Most patients had lepromatous leprosy, according to the Ridley-Jopling classification, four patients had neuropathies, one patient had sequelae (bone deformities in hands and feet) and four patients had leprosy reactions.

**Table 2:** Main characteristics of patients with chromoblastomycosis-leprosy co-infection

General characterístics	*n* (%)	Leprosy characteristics	*n* (%)	Chromoblastomycosis characteristics	*n* (%)
Cases	19	Classification (Ridley–Jopling)		Clinical form	
Distribution by country		Tuberculoid (TT)	0	Verrucous	4 (21.1)
Brazil	15 (78.9)	Borderline tuberculoid (BT)	1 (5.3)	Nodular	3 (15.8)
India	3 (15.8)	Borderline (BB)	3 (15.8)	Cicatricial	4 (21.1)
Japan	1 (5.3)	Borderline lepromatous (BL)	1 (5.3)	Tumorous	1 (5.3)
Gender		Lepromatous (LL)	6 (65.1)	Plaque	1 (5.3)
Male	11 (57.9)	Not described	8 (42.1)	Not described	6 (31.5)
Female	1 (5.3)	Patients with skin lesions	12 (63.2)	Severity	
Not described	7 (36.8)	Patients with neuropathy§	4 (21.1)	Mild	4 (21.1)
Mean ages (range)	52.2 ys (24–91 ys)*	Patients with sequelae	1 (5.3)	Moderate	3 (15.8)
Occupation		Patients with leprosy reactions		Severe	5 (26.3)
Farmer	6 (65.1)	Type I	1 (5.3)	Not described	7 (36.8)
Ironmonger	1 (5.3)	Type II	4 (21.1)	Etiological agent	
Retired	1 (5.3)	No leprosy reactions/Not described	15 (78.9)	*Fonsecaea pedrosoi*	8 (42.1)
Not described	11 (57.9)			*Cladophialophora carrionii*	1 (5.3)
Disease that started first				*Phialopora verrucosa*	1 (5.3)
Leprosy	12 (63.2)			Not described	9 (47,3)
Chromoblastomycosis	7 (36.8)				
Patients with immunosuppressive treatment before the diagnosis of one of the two diseases ‡	5 (26.3)				

*Age reported only in 11 patients. ‡The 5 patients had a previous diagnosis of leprosy and were under steroid treatment for leprosy reactions. §All patients also had skin lesions

In patients with previous onset of chromoblastomycosis, the average time of evolution of this disease until the diagnosis of leprosy was 18.6 years (SD ± 7.6 years; median 21 years; range 5–28 years). The patients presented mainly verrucous and cicatricial lesions and the disease was classified as severe in five of them. The main etiologic agent identified was *F. pedrosoi.*

No close contact or contact with leprosy patients, contact with armadillos or history of lesions at the anatomical site of chromoblastomycosis lesions were reported in the previously and presently reported cases. Five leprosy patients, who were receiving steroid treatment for leprosy reactions or neuropathies, were diagnosed with chromoblastomycosis during immunosuppressive therapy. Table 3 describes the main characteristics of the published cases of chromoblastomycosis-leprosy co-infection.

**Table 3:** Summary of chromoblastomycosis-leprosy co-infection reported cases

References	Country	Age/Sex	Relevant clinical data
[27]	Brazil	66/M	Patient with chromoblastomycosis on the back and palm of the right hand and right wrist, 20 years of evolution with later diagnosis of lepromatous leprosy with type 2 leprosy reaction and basal cell carcinoma of the face and neck
[27]	Brazil	47/M	Patient with chromoblastomycosis in the right leg and foot, 18 years of evolution, later diagnosed with lepromatous leprosy
[41]	Brazil	–/M	Patient with chromoblastomycosis in the left leg and foot, 22 years of evolution with later diagnosis of lepromatous leprosy
[42]	Brazil	32/M	Patient with leprosy, 18 years of evolution, with sequelae of atrophy and bone deformities in the limbs, with later diagnosis of chromoblastomycosis
[43]	Japan	–/–	Patient with lepromatous leprosy who presented a verrucous lesion of chromoblastomycosis in an area of infiltration of a lepromatous lesion in the right leg
[44]	Brazil	50/M	Patient with chromoblastomycosis, 22 years of evolution and later diagnosis of lepromatous leprosy
[28]	Brazil	67/M	Patient with chromoblastomycosis, 21 years of evolution with later diagnosis of borderline tuberculoid leprosy
[45]	India	24/M	Patient with borderline lepromatous leprosy and recurrent type 2 leprosy reactions treated with prednisolone and azathioprine who presented papular, verrucous lesions of chromoblastomycosis in tattoos performed during immunosuppressive treatment
[46]	Brazil	45/M	A patient with leprosy and leprosy reaction treated with prednisone for more than 1 year who presented a verrucous lesion of chromoblastomycosis in the upper limb several months after starting the treatment with corticosteroids
[24]	Brazil	28/M	Patient with multibacillary leprosy, neuritis and type 2 leprosy reaction episodes treated with prednisone and thalidomide. During treatment, he had an erythematous, squamous chromoblastomycosis lesion in his right hand. Four months later, he presented nodular lesions on the trunk, being diagnosed as mucormycosis
[8]*	India	–/–	Patients with leprosy and treatment with corticosteroids later diagnosed with chromoblastomycosis. Patients had long-term corticosteroid treatment
[12]**	Brazil	–/–	Leprosy patients who were later diagnosed with chromoblastomycosis. No previous corticosteroid treatment

*Two patients with chromoblastomycosis-leprosy co-infection reported in a series of chromoblastomycosis cases. **Four patients with chromoblastomycosis-leprosy co-infection reported in a series of chromoblastomycosis cases

## Discussion

Chromoblastomycosis and leprosy are chronic diseases that affect a large number of individuals who predominantly live in tropical and subtropical regions, affecting the poorest and most vulnerable populations. The World Health Organization has classified the two diseases within the group of Neglected Tropical Diseases (NTDs) and has coordinated worldwide efforts for their respective control and elimination [11].

Cases of chromoblastomycosis-leprosy co-infection have rarely been reported and are mostly from Brazil. When observing the epidemiological characteristics of patients with chromoblastomycosis-leprosy co-infection, we can find common aspects present in both co-infection and in the two diseases separately: male predominance, all age groups are affected, occupation and origin mainly related to the rural area, poverty, living conditions, inequity, difficulty in accessing public health services. Factors that are present in different countries in tropical and subtropical regions where these diseases are endemic [1, 4, 5, 13, 17–23].

There is no predisposing relationship from one disease to another, except for prolonged treatment with corticosteroids in leprosy reactions or other complications of this disease, which can cause immunosuppression and predispose patients to other infectious diseases such as chromoblastomycosis, as described in the various cases published. The explanation of co-infection could be attributed to the epidemiology of both diseases. Brazil is one of the countries with the highest incidence and prevalence of chromoblastomycosis and leprosy, thus co-infection can be expected in some patients [1–3, 12, 13]. Co-infection should be especially considered if a patient: (i) resides or works in a rural area; (ii) is exposed to the habitats of the etiological agents of chromoblastomycosis; (iii) is within the habitat of armadillos (*Dasypus* species) that are known to be zoonotic reservoirs of leprosy [1, 2, 9, 24, 25]. Most cases of chromoblastomycosis associated with leprosy, including the three cases reported here, were caused by *F. pedrosoi*, considered the main etiologic agent of the disease in Brazil [1, 12, 14, 15, 26].

An interesting aspect of the reported cases of chromoblastomycosis-leprosy co-infection is its occurrence in elderly patients. Only two patients described in previous publications were elderly of ≥ 65 years of age, in the current study 2 out of 3 patients fell in this age category [27, 28]. Altogether, these represent 21.1% of reported cases of chromoblastomycosis-leprosy co-infection.

Both chromoblastomycosis and leprosy, separately, can affect the elderly. Recent studies about the epidemiology of chromoblastomycosis have reported that the average age is above 52 years [5, 13]. While studies on the epidemiology of leprosy found an increase in the detection of this disease in the elderly when compared to other age groups [20, 29, 30]. Therefore, co-infection in elderly is possible especially in areas endemic for both diseases.

The explanation for the occurrence of chromoblastomycosis-leprosy co-infection in the elderly patients may be complex. In addition to epidemiological factors and drug-induced immunosuppression, it could be related to immunosenescence, which is the process of immune dysfunction that occurs with age and includes lymphoid tissue remodeling and changes in the immune system of the elderly, making them more susceptible to the development of infectious diseases, autoimmune diseases and cancer [31–33].

The innate immune response is very important in both chromoblastomycosis and leprosy [34–36]. In immunosenescence, dysfunction of innate immunity cells is observed, causing a decrease in phagocytosis, destruction of fungal elements and an increase in the immunomodulation produced by the etiological agents of leprosy, facilitating the progression of both diseases. The alteration of adaptive immunity by immunosenescence is characterized by the imbalance in the TCD4+ response, with a predominance of the Th17 lymphocyte response over the regulatory T (Treg), which is also stimulated by the etiologic agents of chromoblastomycosis, producing an inefficient immune response characterized by chronic inflammation [37, 38]. Little is known about the Th17 response in the elderly with leprosy, but this response has been associated with tuberculoid leprosy [35, 39, 40]. The Th17 response works together with the cells of innate immunity and the Th1 response, which are limited in immunosenescence, therefore, it is possible that the Th17 response is not sufficient to prevent the progression of leprosy in the elderly. The synergy between immunosenescence and the changes induced by the etiological agents of both chromoblastomycosis, and leprosy stimulates an immune response that is unable to contain both diseases, favoring their progression.

## Conclusions

Chromoblastomycosis and leprosy co-infections are uncommon, but the possibility should always be considered, especially if the patient is undergoing immunosuppressive treatment or is elderly. The reported cases of chromoblastomycosis-leprosy co-infection demonstrate the importance of knowledge about the epidemiology of infectious diseases that coexist in the same region, which may allow for a more accurate and early diagnosis, and an improvement in the patient's prognosis.
